# Autoimmune Progressive Fibrosing Interstitial Lung Disease: Predictors of Fast Decline

**DOI:** 10.3389/fphar.2021.778649

**Published:** 2021-12-22

**Authors:** Alexandra Nagy, Tamas Nagy, Abigel Margit Kolonics-Farkas, Noemi Eszes, Krisztina Vincze, Eniko Barczi, Adam Domonkos Tarnoki, David Laszlo Tarnoki, György Nagy, Emese Kiss, Pal Maurovich-Horvat, Aniko Bohacs, Veronika Müller

**Affiliations:** ^1^ Department of Pulmonology, Semmelweis University, Budapest, Hungary; ^2^ Medical Imaging Centre, Semmelweis University, Budapest, Hungary; ^3^ Department of Genetics, Cell- and Immunobiology, Semmelweis University, Budapest, Hungary; ^4^ Department of Rheumatology and Clinical Immunology, Semmelweis University, Budapest, Hungary; ^5^ Department of Clinical Immunology, Adult and Pediatric Rheumatology, National Institute of Locomotor Diseases and Disabilities, Budapest, Hungary; ^6^ 3rd Department of Internal Medicine and Haematology, Semmelweis University, Budapest, Hungary

**Keywords:** autoimmune disease, progressive fibrosing interstitial lung disease (PF-ILD), connective tissue disease (CTD), interstitial pneumonia with autoimmune features (IPAF), treatment, antifibrotics

## Abstract

A subset of interstitial lung diseases (ILDs) with autoimmune traits—including connective tissue disease-associated ILD (CTD-ILD) and interstitial pneumonia with autoimmune features (IPAF)—develops progressive fibrosing (PF)-ILD. The aim of our study was to evaluate the clinical characteristics and predictors of longitudinal lung function (LF) changes in autoimmune PF-ILD patients in a real-world setting. All ILD cases with confirmed or suspected autoimmunity discussed by a multidisciplinary team (MDT) between January 2017 and June 2019 (*n* = 511) were reviewed, including 63 CTD-ILD and 44 IPAF patients. Detailed medical history, LF test, diffusing capacity of the lung for carbon monoxide (DLCO), 6-min walk test (6MWT), blood gas analysis (BGA), and high-resolution computer tomography (HRCT) were performed. Longitudinal follow-up for functional parameters was at least 2 years. Women were overrepresented (70.1%), and the age of the IPAF group was significantly higher as compared to the CTD-ILD group (*p* < 0.001). Dyspnea, crackles, and weight loss were significantly more common in the IPAF group as compared to the CTD-ILD group (84.1% vs. 58.7%, *p* = 0.006; 72.7% vs. 49.2%, *p* = 0.017; 29.6% vs. 4.8%, *p* = 0.001). Forced vital capacity (FVC) yearly decline was more pronounced in IPAF (53.1 ± 0.3 vs. 16.7 ± 0.2 ml; *p* = 0.294), while the majority of patients (IPAF: 68% and CTD-ILD 82%) did not deteriorate. Factors influencing progression included malignancy as a comorbidity, anti-SS-A antibodies, and post-exercise pulse increase at 6MWT. Antifibrotic therapy was administered significantly more often in IPAF as compared to CTD-ILD patients (*n* = 13, 29.5% vs. *n* = 5, 7.9%; *p* = 0.007), and importantly, this treatment reduced lung function decline when compared to non-treated patients. Majority of patients improved or were stable regarding lung function, and autoimmune-associated PF-ILD was more common in patients having IPAF. Functional decline predictors were anti-SS-A antibodies and marked post-exercise pulse increase at 6MWT. Antifibrotic treatments reduced progression in progressive fibrosing CTD-ILD and IPAF, emphasizing the need for guidelines including optimal treatment start and combination therapies in this special patient group.

## Introduction

Interstitial lung diseases (ILDs) are a heterogeneous group of lung disorders, with diffuse parenchymal involvement also associated with a relevant morbidity and mortality. The spectrum of ILD is very diverse and the etiology is often idiopathic; however, a significant proportion of patients present with confirmed or possible autoimmune characteristics (Antoniou et al., 2014; Fischer et al., 2015; Martin et al., 2016). Connective tissue diseases (CTDs) are often associated with ILD. Lung involvement may occur in the initial phase of the systemic autoimmune disorder; however, ILD can manifest even before the diagnosis of CTD (Fischer and Du Bois, 2012). The term “interstitial pneumonia with autoimmune features” (IPAF) describes a type of interstitial pneumonias that are clinically and serologically associated with autoimmune characteristics but do not correspond completely to the diagnostic criteria of CTD (Sambataro et al., 2018).

Continuous monitoring of patients is essential to recognize progression (Fisher et al., 2020). The phenotype of progressive fibrosing (PF)-ILD, regardless of the underlying disease, shows common clinical features of lung function decline (Johannson et al., 2021). Worsening of symptoms—mainly dyspnea and cough—is often associated with progression of fibrosis on high-resolution computer tomography (HRCT); however, the definition for PF-ILD is not unitary (Cottin et al., 2018; Cottin et al., 2019; Brown et al., 2020; Kolb and Flaherty, 2021). PF-ILD results in the deterioration of quality of life and leads to early mortality. Forced vital capacity (FVC) and diffusing capacity of the lung for carbon monoxide (DLCO) decline are important and most frequently accepted markers of progression and are predictive factors of mortality (Brown et al., 2020), (Volkmann et al., 2019; Solomon et al., 2016; George et al., 2020).

A multidisciplinary approach is crucial for proper ILD diagnosis and treatment (Grewal et al., 2019). Considering the wide spectrum of disorders among autoimmune ILDs including different CTD-ILDs and even IPAF, it is essential to outline the best therapeutic possibilities for these patients. In addition to immunosuppressive therapy being extensively used, new antifibrotic agents—nintedanib and pirfenidone—also impact on the disease course; however, data on the interaction between these medications are lacking (Johannson et al., 2021; Wollin et al., 2019; Maher et al., 2020; Gao and Moua, 2020). It is challenging to find the best time for the introduction of certain drugs as well as choosing the optimal treatment course and combination for autoimmune-mediated ILDs (Cottin et al., 2019; Flaherty et al., 2019; George et al., 2020).

Our goal was to assess the clinical course of autoimmune ILDs—regarding the PF-ILD phenotype—and to confirm risk factors for progression and potential beneficial therapies in a real-word setting.

## Materials and Methods

### Study Population

Our study is based on retrospective data analysis of ILD patients. Each case was presented and diagnosed by our multidisciplinary team (MDT) including pulmonologists, rheumatologists, radiologists, and pathologists. The ILD-MDT evaluation of the patients was performed at the Department of Pulmonology Semmelweis University between January 2017 and June 2019 (Richeldi et al., 2019).

The diagnosis of CTD was based on the internationally accepted American College of Rheumatology/European League Against Rheumatism Collaborative Initiative (EULAR-ACR) clinical and serologic criteria by rheumatology specialists working at one of the two rheumatology centers in Central Hungary. CTDs included rheumatoid arthritis (RA), systemic sclerosis (SSc), systemic lupus erythematosus (SLE), vasculitis, idiopathic inflammatory myopathies including polymyositis/dermatomyositis (IIM; PM/DM), and other categories [mixed connective tissue disease (MCTD) and undifferentiated connective tissue disease (UCTD)] (Kay and Upchurch, 2012; Van Den Hoogen et al., 2013; Petri et al., 2012; Aringer, 2019; Yates et al., 2016; Lundberg et al., 2017; Ortega-Hernandez and Shoenfeld, 2012; Mosca et al., 2014). The diagnosis of IPAF consisted of clinical, serological, and morphological domains based on the classification criteria proposed by the European Respiratory Society/American Thoracic Society (ERS/ATS) in 2015 (Fischer et al., 2015). All patients were consulted by rheumatologists to exclude manifestations of CTD at the time of diagnosis or in case of clinical suspicion thereafter. None of the IPAF patients developed CTD during follow-up.

At baseline and every follow-up, physical examination was performed, and a detailed medical history was taken with special emphasis on symptoms (dry/productive cough, sputum, and chest pain), respiratory infections, and comorbidities (Barczi et al., 2020). In our clinical routine, studied autoantibodies were anti-nuclear antibodies (ANA), rheumatoid factor (RF), anti-cyclic citrullinated peptide antibodies (ACPA), anti-RNA polymerase, anti-centromere, anti-proliferating cell nuclear antigen (APCNA), anti-Ku, anti-P-ribosomal, anti-cytoplasmatic, anti-cytoskeleton, anti-chromatin, anti-Smith, anti-myeloperoxidase, anti-proteinase-3, anti-Jo-1, anti-SS-A, anti-SS-B, anti-SCL-70, anti-ribonucleoprotein (RNP), and anti-neutrophil cytoplasmic antibodies (ANCA).

Baseline lung HRCT scans, pulmonary function test (PFT), blood gas analysis (BGA), and 6-min walk test (6MWT) were implemented at the time of the ILD diagnosis. Gender-age-physiology (GAP) score was used for clinical severity prediction in CTD-ILD and IPAF (Ryerson et al., 2014).

**FIGURE 1:**
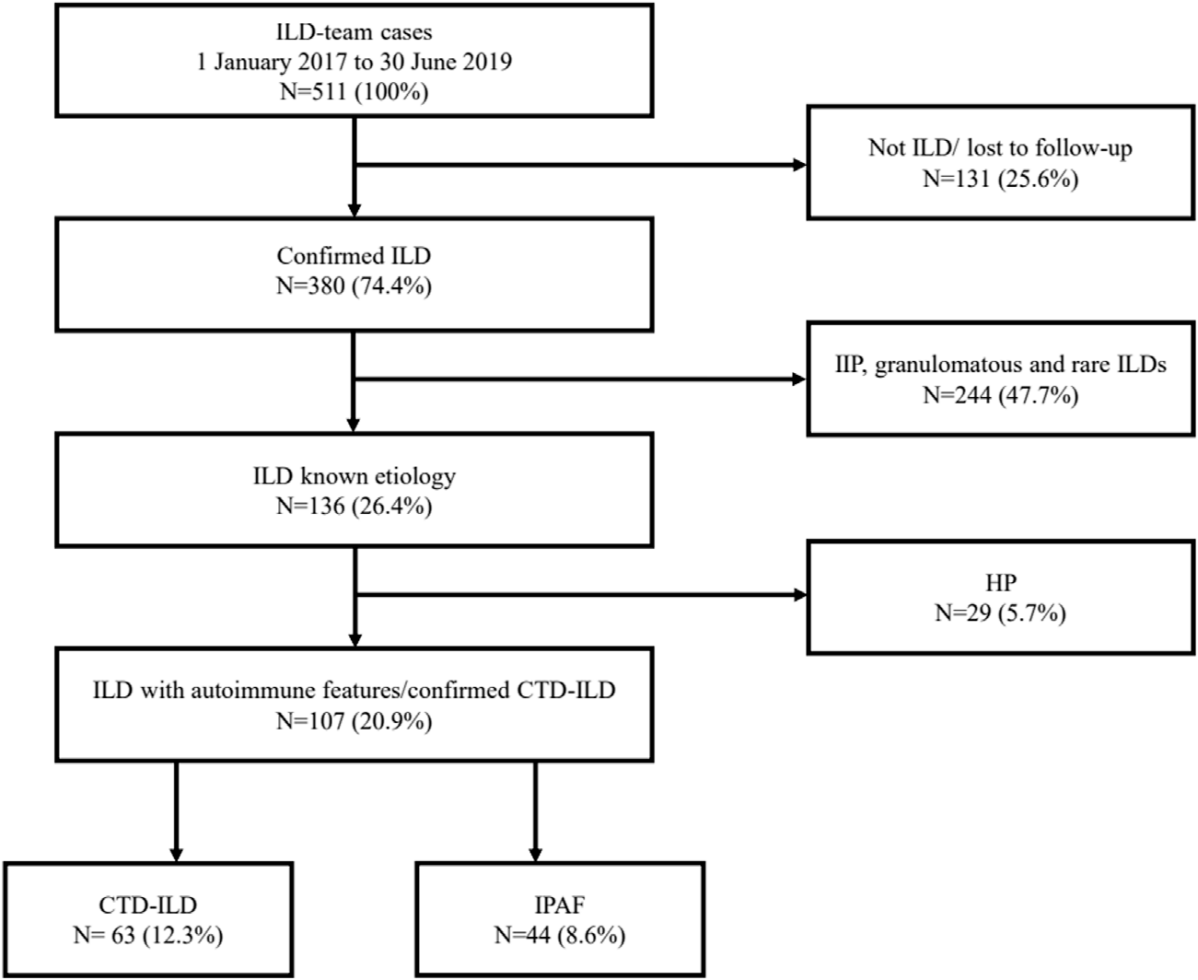
Study population. ILD, interstitial lung disease; IIP, idiopathic interstitial pneumonia; HP, hypersensitivity pneumonitis; CTD-ILD, connective tissue disease-associated interstitial lung disease; IPAF, interstitial pneumonia with autoimmune features.

Confirmed ILDs were classified into four main groups: ILDs with known etiology including mainly confirmed CTD-ILD and hypersensitive pneumonitis (HP) cases; idiopathic interstitial pneumonia (IIP) including idiopathic pulmonary fibrosis (IPF), idiopathic non-specific interstitial pneumonia (iNSIP), and other IIPs; granulomatous diseases; and other rare forms of ILDs according to current guidelines (Travis et al., 2013), (Raghu et al., 2011). IPAF was considered a separate entity; nevertheless, it was included in the first group. The study population selection is summarized in Figure 1. Patients with autoimmune characteristics were divided into two subgroups: CTD-ILD and IPAF patients.

The long-term care included pulmonary and rheumatology controls defined by patients’ disease requirements.

### Pulmonary Evaluation and Functional Measurements and Radiological Patterns

PFT, including the analysis of FVC, forced expiratory volume in 1 s (FEV1), FEV1/FVC, total lung capacity (TLC), was performed according to the standardized protocol at the Department of Pulmonology. Lung diffusion capacity was measured for DLCO using the single-breath CO method, and transfer coefficient of the lung for CO (KLCO) was calculated (PDD-301/s, Piston, Budapest, Hungary). Exercise tolerance was established using the 6MWT. Distance in meters (m), baseline and post-exercise oxygen saturation (SpO_2_), heart rate, and Borg scale referring to dyspnea were assessed. Arterialized capillary BGA were evaluated at room air temperature (Cobas b 221, Roche, Hungary).

HRCT scan was performed in both inspiration and expiration positions using Philips Ingenuity Core 64 and Philips Brilliance 16 CT scanners. NSIP pattern was divided into cellular and fibrotic subtypes by radiologist experts according to HRCT scans. Radiologic features typically include cellular variant with ground-glass opacities and fine reticular opacities; besides, the fibrotic subtype is characterized predominantly by traction bronchiectasis (Kligerman et al., 2009). In case of usual interstitial pneumonia (UIP), honeycombing with subpleural and basal predominance can be observed. Traction bronchiectasis might be associated with ground-glass opacification. The pattern of probable UIP (pUIP) is characterized by the same abnormalities without honeycombing (Raghu et al., 2011).

Pulmonary follow-up of at least 24 months after ILD diagnosis included measurements of lung function parameters, diffusion capacity, laboratory testing, and BGA controls. At this time point, we recorded the immunosuppressive and/or antifibrotic therapies between the visit intervals. All CTD patients were followed at the respective rheumatology centers.

In our study, PF-ILD was defined as FVC relative yearly decline ≥5% and either deterioration of clinical symptoms or progression of fibrosis on HRCT (Cottin et al., 2018).

### Statistical Analysis

Analysis was performed using the GraphPad software (GraphPad Prism 5.0 Software, Inc., La Jolla, CA, United States) and SPSS v25 (IBM Corporation, Armonk, NY, United States). Continuous variables were expressed as mean ± standard deviation. Normality of the data was determined using Kolmogorov–Smirnov test. Differences between groups for continuous data were evaluated in normally distributed data with Student’s *t*-test; otherwise, Mann–Whitney U-test was used. Chi-squared test and two-tailed Fisher’s exact test were applied for comparing categorical variables. Predictors of progression were analyzed using Cox proportional hazards regression model. All percentage values are expressed for the whole study population or respective subgroups as indicated. A *p*-value <0.05 was defined as statistically significant.

## Results

Patient characteristics are summarized in Table 1. The study population included mainly women. Patients in the IPAF subgroup were significantly older compared to the CTD subgroup. Dyspnea, crackles, and weight loss were significantly more common in the IPAF group as compared to the CTD-ILD group. Subtypes of CTD (*n* = 63) were, by order of prevalence, SSc (50.8%) RA (20.6%), SLE (9.5%), others (MCTD and UCTD) (9.5%), PM/DM (6.4%), and vasculitis (3.2%). Raynaud’s phenomenon occurred significantly more often in patients with known CTD. LF at baseline is summarized in Table 2. Patients were characterized by mild restrictive functional impairment. There was a slight decrease in TLC and CO diffusion parameters. No differences in LF, 6MWT, or BGA were noted between the two groups.

**TABLE 1:** Patient characteristics.

Parameters	All patients (*n* = 107)	CTD-ILD (*n* = 63)	IPAF (*n* = 44)	*p*-value
Age (years)	63.78 ± 13.88	59.73 ± 14.08	69.57 ± 11.45	**<0.001**
Sex (male/female), *n*	32:75	13:50	19:25	**0.018**
Ever smoker, *n* (%)	44 (41.12)	22 (34.92)	22 (50.0)	0.162
Non-smoker, *n* (%)	63 (58.87)	41 (65.08)	22 (50.0)	
BMI (kg/m^2^)	25.60 ± 6.22	25.87 ± 4.83	25.27 ± 7.10	0.604
Symptoms, *n* (%)	–	–	–	–
Dyspnea	74 (69.16)	37 (58.73)	37 (84.09)	**0.006**
Cough	63 (58.57)	34 (53.97)	29 (65.91)	0.237
Dry cough	38 (35.51)	19 (30.16)	19 (43.18)	0.218
Sputum	25 (23.36)	15 (23.81)	10 (22.73)	1.000
Chest pain	20 (18.69)	10 (15.87)	10 (22.73)	0.452
Joint pain	57 (53.27)	36 (57.14)	21 (47.73)	0.431
Clubbing	12 (11.21)	4 (6.35)	8 (18.18)	0.068
Weight loss	16 (14.95)	3 (4.76)	13 (29.55)	**0.001**
Crackles	63 (58.88)	31 (49.21)	32 (72.73)	**0.017**
Raynaud’s phenomenon	32 (29.91)	27 (42.86)	5 (11.36)	**<0.001**
CTD subtype, *n* (%)	–	–	–	–
RA	–	13 (20.63)	–	–
SSc	–	32 (50.79)	–	–
SLE	–	6 (9.52)	–	–
Vasculitis	–	2 (3.17)	–	–
DM/PM	–	4 (6.35)	–	–
Others (MCTD and UCTD)	–	6 (9.52)	–	–

CTD-ILD, connective tissue disease-associated interstitial lung disease; IPAF, interstitial pneumonia with autoimmune features; BMI, body mass index; RA, rheumatoid arthritis; SSc, systemic sclerosis; SLE, systemic lupus erythematosus; PM/DM, polymyositis/dermatomyositis; MCTD, mixed connective tissue disease; UCTD, undifferentiated connective tissue disease.

Statistically significant values were highlighted with bold in the tables.

**TABLE 2:** Functional parameters.

Parameters	All patients (*n* = 107)	CTD-ILD (*n* = 63)	IPAF (*n* = 44)	*p*-value
Lung function	–	–	–	–
FEV1/FVC	0.84 ± 0.08	0.84 ± 0.06	0.82 ± 0.10	0.287
FVC (L)	2.50 ± 0.86	2.49 ± 0.89	2.52 ± 0.83	0.951
FVC (%)	84.41 ± 23.86	85.51 ± 26.93	82.82 ± 18.72	0.577
FEV1 (L)	2.08 ± 0.72	2.09 ± 0.73	2.07 ± 0.71	0.819
FEV1 (%)	85.64 ± 24.67	86.82 ± 26.26	83.93 ± 22.36	0.562
TLC (L)	4.31 ± 1.43	4.39 ± 1.54	4.19 ± 1.26	0.683
TLC (%)	80.64 ± 24.82	83.86 ± 26.54	76.13 ± 21.73	0.133
Diffusion parameters	–	–	–	–
DLCO (mmol/min/kPa)	5.52 ± 1.87	5.55 ± 1.84	5.47 ± 1.94	0.899
DLCO (%)	70.92 ± 20.88	70.53 ± 20.07	71.48 ± 22.21	0.823
KLCO (mmol/min/kPa/l)	1.26 ± 0.38	1.27 ± 0.37	1.24 ± 0.39	0.943
KLCO (%)	66.19 ± 18.54	65.25 ± 18.12	67.50 ± 19.26	0.551
BGA	–	–	–	–
pH	7.42 ± 0.04	7.43 ± 0.05	7.42 ± 0.02	0.204
pCO2	40.10 ± 11.13	41.13 ± 11.87	38.86 ± 10.19	0.859
pO2	66.69 ± 11.82	65.63 ± 13.85	67.96 ± 8.80	0.859
6MWT	–	–	–	–
Distance (m)	400.73 ± 108.15	403.45 ± 120.96	397.61 ± 93.02	0.822
SpO_2_ baseline	94.51 ± 4.15	95.00 ± 3.35	93.91 ± 4.94	0.490
SpO_2_ post-exercise	90.12 ± 8.97	90.69 ± 6.74	89.47 ± 11.06	0.223
Pulse baseline	84.05 ± 14.50	84.75 ± 12.88	83.24 ± 16.37	0.658
Pulse post-exercise	106.71 ± 19.83	109.84 ± 19.56	103.21 ± 19.82	0.158
Borg scale baseline	2.01 ± 11.46	3.23 ± 15.42	0.55 ± 1.25	0.253
Borg scale post-exercise	4.05 ± 11.05	5.33 ± 14.86	2.56 ± 2.15	0.223

CTD-ILD, connective tissue disease-associated interstitial lung disease; IPAF, interstitial pneumonia with autoimmune features; FVC, forced vital capacity; FEV1, forced expiratory volume in 1 s; TLC, total lung capacity; DLCO, diffusing capacity for carbon monoxide; KLCO, transfer coefficient of the lung for carbon monoxide; BGA, blood gas analysis; 6MWT, 6-min walk test.

The most common radiological pattern was NSIP; however, significantly more pUIP was noted in IPAF patients. HRCT data are summarized in Table 3. Most frequently confirmed auto-antibodies were ANA, followed by anti-chromatin antibodies and RF, with no differences among the two groups (Table 4).

**TABLE 3:** HRCT morphological domain.

HRCT pattern	All patients (*n* = 107)	CTD-ILD (*n* = 63)	IPAF (*n* = 44)	*p*-value
pUIP, *n* (%)	27 (25.23)	8 (12.70)	19 (43.18)	**0.001**
UIP, *n* (%)	20 (18.69)	10 (15.87)	10 (22.73)	0.370
NSIP, *n* (%)	46 (42.99)	38 (60.32)	8 (18.18)	**<0.001**

HRTC, high-resolution computed tomography; CTD-ILD, connective tissue disease-associated interstitial lung disease; IPAF, interstitial pneumonia with autoimmune features; pUIP, probable usual interstitial pneumonia; UIP, usual interstitial pneumonia; NSIP, non-specific interstitial pneumonia.

Statistically significant values were highlighted with bold in the tables.

**TABLE 4:** Autoimmune serology.

Autoantibodies	All patients (*n* = 107)	CTD-ILD (*n* = 63)	IPAF (*n* = 44)	*p*-value
ANA, *n* (%)	71 (66.36)	43 (68.25)	28 (63.64)	0.330
RF, *n* (%)	22 (20.56)	11 (17.46)	11 (25.00)	0.466
ACPA, *n* (%)	10 (9.35)	5 (7.94)	5 (11.36)	0.738
Anti-RNA-polymerase, *n* (%)	0	0	0	–
Anti-centromere, *n* (%)	1 (0.93)	1 (1.59)	0	–
Anti-PCNA, *n* (%)	2 (1.87)	1 (1.59)	1 (2.27)	1.000
Anti-Ku, *n* (%)	0	0	0	0
Anti-P-ribosomal, *n* (%)	0	0	0	0
Anti-cytoplasmatic, *n* (%)	27 (25.23)	17 (26.98)	10 (22.73)	0.658
Anti-cytoskeleton, *n* (%)	0	0	0	0
Anti-chromatin, *n* (%)	32 (29.90)	19 (30.16)	13 (29.55)	1.000
Anti-Smith, *n* (%)	4 (3.73)	2 (3.17)	2 (4.55)	1.000
Anti-myeloperoxidase, *n* (%)	2 (1.87)	2 (3.17)	0	–
Anti-proteinase-3, *n* (%)	1 (0.93)	1 (1.59)	0	–
Anti-Jo-1, *n* (%)	3 (2.80)	2 (3.17)	1 (2.27)	1.000
Anti-SS-A, *n* (%)	18 (16.82)	12 (19.05)	6 (13.64)	0.602
Anti-SS-B, *n* (%)	5 (4.67)	3 (4.76)	2 (4.55)	1.000
Anti-SCL-70, *n* (%)	17 (15.88)	17 (26.98)	0	–
Anti-RNP, *n* (%)	10 (9.34)	8 (12.70)	2 (4.55)	0.192
ANCA, *n* (%)	8 (7.48)	4 (6.35)	4 (9.09)	0.714

CTD-ILD, connective tissue disease-associated interstitial lung disease; IPAF, interstitial pneumonia with autoimmune features; ANA, anti-nuclear antibodies; RF, rheumatoid factor; ACPA, anti-cyclic citrullinated peptide antibodies; APCNA, anti-proliferating cell nuclear antigen; ANCA, anti-neutrophil cytoplasmic antibodies.

Fifty-nine patients had functional data during the 24-month follow-up including 34 CTD-ILD (23.5% males; mean age 58.42 ± 13.01 years) and 25 IPAF (48.0% males; mean age 69.02 ± 12.51 years) patients. Baseline data of CTD-ILD [SSc (55.9%), RA (20.6%), PM/DM (11.8%), SLE (5.9%), and other MCTD and UCTD (5.9%)] and IPAF patients with available functional follow-up did not differ in any parameter from the whole respective group. To estimate mortality, we applied the GAP risk prediction model, which is also validated for non-IPF ILDs (Ryerson et al., 2014). Values were markedly better in the CTD group compared to the IPAF group (1.82 vs. 2.48, *p* = 0.07).

**FIGURE 2:**
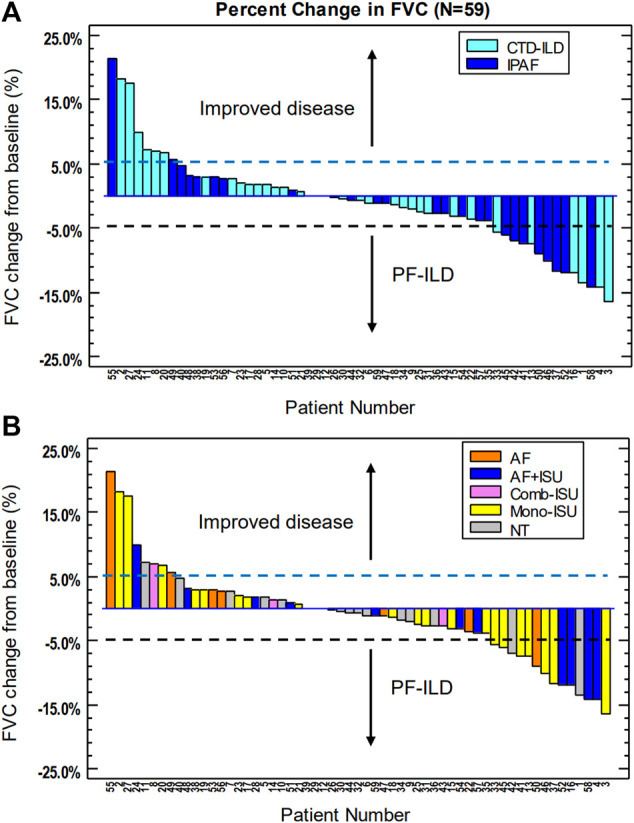
Longitudinal follow-up of CTD-ILD and IPAF patients: percent change in FVC. **(A)** Changes according to treatment; **(B)** respective patients according to underlying disease. CTD-ILD, connective tissue disease-associated interstitial lung disease; IPAF, interstitial pneumonia with autoimmune features; FVC, forced vital capacity; PF-ILD, [progressive fibrosing ILD; AF, antifibrotic treatment; AF + ISU, antifibrotic treatment with immunosuppressive agent; Comb-ISU, combined immunosuppressive treatment; Mono-ISU, one immunosuppressive agent; NT, no treatment].

FVC yearly decline was more dominant in the IPAF group than in the CTD-ILD group (53.1 ± 0.3 ml vs. 16.7 ± 0.2 ml; *p* = 0.294) (Figure 2A). It is important to note that 68.0% (out of the followed 25 patients) did not deteriorate in the IPAF group as compared to 82.4% (out of followed 34 patients) in the CTD-ILD group (*p* = 0.200). PF-ILD criteria were met by 14 patients. We also determined the prevalence of PF-ILD in each entity of CTD-ILD: RA (*n* = 3), SSc (*n* = 2), other (*n* = 1), and IPAF (*n* = 8).

Factors influencing rapid progression qualifying as PF-ILD included malignancy as a comorbidity, ANA, anti-SS-A antibodies, and post-exercise pulse increase at the 6MWT (Table 5). Malignancy was diagnosed in seven patients (two males and five females) including CML (1), lung (2), ovarian (1), breast (1), esophageal (1), and laryngeal cancer (1). There was no correlation between HRCT pattern (UIP, pUIP, fibrotic, or cellular NSIP) and progression. Detailed data were not included, as no relationship was present.

**TABLE 5:** Factors influencing progression of autoimmune ILDs.

Factor	HR	95% CI	*p*-value
Patient comorbidities	–	–	–
Hypertension	1.27	0.34 to 4.68	0.721
Thyroid disorder	11.90	0.77 to 182.80	0.076
Malignancy	8.17	1.31 to 50.81	**0.024**
PAH	1.52	0.34 to 6.84	0.584
Smoking	1.11	0.26 to 4.70	0.891
BMI	0.92	0.80 to 1.05	0.21
BGA	–	–	–
pH	21.82	0.00	0.936
pCO_2_	0.10	0.83 to 1.20	0.990
pO_2_	0.93	0.80 to 1.09	0.366
6MWT	–	–	–
Distance (m)	0.99	0.98 to 1.01	0.309
SpO_2_ baseline	1.69	0.79 to 3.60	0.173
SpO_2_ post-exercise	0.87	0.61 to 1.26	0.474
Pulse baseline	0.98	0.87 to 1.11	0.783
Pulse post-exercise	1.14	1.00 to 1.29	**0.043**
Borg scale baseline	0.72	0.12 to 4.37	0.722
Borg scale post-exercise	0.64	0.22 to 1.84	0.403
HRCT pattern	1.20	0.58 to 2.48	0.632
Autoantibodies	–	–	–
ANA	0.13	0.02 to 0.92	**0.041**
RF	3.27	0.23 to 45.95	0.380
ACPA	1.55	0.13 to 18.23	0.730
Anti-PCNA	0.00	0.00	0.992
Anti-cytoplasmatic	5.36	0.60 to 48.12	0.134
Anti-chromatin	0.47	0.08 to 2.83	0.411
Anti-Jo-1	6.13	0.08 to 482.05	0.416
Anti-SS-A	13.11	1.71 to 100.45	**0.013**
Anti-SS-B	2.23	0.02 to 279.51	0.745
Anti-SCL-70	0.97	0.07 to 12.94	0.980
Anti-RNP N	2.08	0.16 to 27.61	0.579
ANCA	0.00	0.00	0.997

HR, hazard ratio; CI, confidence interval; PAH, pulmonary arterial hypertension; BMI, body mass index; BGA, blood gas analysis; 6MWT, 6-min walk test; HRTC, high-resolution computed tomography; ANA, anti-nuclear antibodies; RF, rheumatoid factor; ACPA, anti-cyclic citrullinated peptide antibodies; APCNA, anti-proliferating cell nuclear antigen; ANCA, anti-neutrophil cytoplasmic antibodies.

Statistically significant values were highlighted with bold in the tables.

During the follow-up period, 16 patients (CTD-ILD *n* = 11; IPAF *n* = 5) did not receive any treatment. Conventional immunosuppressive (ISU) therapies including corticosteroids, rituximab, mycophenolate mofetil, azathioprine, cyclophosphamide, and methotrexate were the initial medical treatment in 36 cases (CTD-ILD *n* = 22; IPAF *n* = 14). Mono or combined ISU therapies were appropriate during follow-up period for 25 patients (CTD-ILD *n* = 18; IPAF *n* = 7). In some cases, when antifibrotic therapy was given, progressive phenotype was observed. Patients showing progressive phenotype are those whose ISU therapy was supplemented with antifibrotic therapies such as nintedanib and pirfenidone. Antifibrotic drugs were administered significantly more often in IPAF as compared to CTD-ILD (*n* = 13 vs. *n* = 5; *p* = 0.007). The majority of these patients (72.2% on antifibrotic treatment) represented stable lung function or improvement following treatment introduction. Individual functional change according to therapy is summarized in Figure 2B. Antifibrotic treatment (pirfenidone 801 mg tid *n* = 2; nintedanib 150 mg bid *n* = 17, including one patient who switched to pirfenidone due to elevated liver enzymes)-related adverse events—all grade 1 and transient—included gastrointestinal symptoms, mainly nausea and vomiting, diarrhea, and heartburn. Most of them were solved by dosage reduction and supportive medications. Elevated liver enzymes were only observed in one patient and was resolved after changing to another antifibrotic drug. Unfortunately, during follow-up, nine patients with mono or combined ISU therapy developed PF-ILD according to our criteria, four (CTD-ILD *n* = 2; IPAF *n* = 2) of them had anti-SS-A antibody positivity and five patients (CTD-ILD *n* = 3; IPAF *n* = 2) had post-exercise pulse increase.

## Discussion

We presented the first single-center real-life data analyzing the functional progression of autoimmune ILDs. A small proportion of CTD-ILD and IPAF patients deteriorated (13.1% of the whole population) over the observed period, which is similar to other international data (Simpson et al., 2021). Most of the patients were stable, and remarkably, eight patients had even ≥5% FVC improvement due to therapy out of 59 followed.

Our data are the first to show ILD distribution of cases from an Eastern European country. Out of the 511 cases presented to the ILD team, 20.9% were CTD-ILD or IPAF, which is very similar to international data (Oldham et al., 2016; Sambataro et al., 2019). CTD-ILD did mainly include SSc (50.8%) and RA (20.6%) patients, also in line with previously published numbers (Oliveira et al., 2020; Sambataro et al., 2018).

IPAF is mainly considered as a research entity with an autoimmune profile and affected 25 patients in our study. Assessment by rheumatology specialists and serological testing were always performed to confirm or exclude CTD in these cases (Fischer et al., 2015; Sambataro et al., 2018; Raghu et al., 2018). However, there is no international agreement on which serological tests are required at the first encounter with the patient (Jee et al., 2017). The serological pattern in IPAF patients was consistent with the current classification criteria (Fischer et al., 2015; Sambataro et al., 2018).

The most common radiological pattern among IPAF patients was pUIP, which correlates with the data of Oldham et al. (2016); however, it contradicts prospective international data where NSIP was the most frequent pattern (Ahmad et al., 2017; Sambataro et al., 2018). In a retrospective study, UIP and non-UIP IPAF had a similar chance to transform into specific autoimmune diseases; thus, the role of the morphological domain of IPAF is questionable (Sambataro et al., 2020). HRCT evaluation is not homogenous among ILD expert radiologists and might have contributed partially to these differences (Walsh et al., 2016), (Widell and Lidén, 2020). Additionally, IPAF is not a homogenous entity, as it may be very similar to CTD-ILD or in contrast to IPF (Oldham et al., 2016; Ferri et al., 2016).

Treatment resulted in lung function improvement, especially in CTD-ILD. Variation of disease course is well known in SSc, where patients can have a rapid progression, stability of disease, and even improvement. Our data confirmed that most patients’ lung function remained stable over the 2-year period; some of them even improved similarly to the Scleroderma Lung Study (SLS) I and II trials and SENSCIS (Volkmann et al., 2017), (Vonk et al., 2021).

An important new finding and interesting consideration of our study is the identification of new possible prognostic factors for PF-ILD in autoimmune-mediated ILDs including ANA and anti-SS-A antibodies, post-exercise pulse increase at 6MWT, and malignancy. Anti-SS-A antibodies such as Ro52 and Ro60 are often used in autoimmune disease diagnosis. Based on literature data, isolated anti-SS-A/Ro60+ is independently associated with SLE. Detection of anti-SS-A/Ro52+ has a prognostic importance in SSc-associated ILD and diagnostic value in PM/DM (Robbins et al., 2019; Hudson et al., 2012; Dugar et al., 2010; Menéndez et al., 2013). Previous small cohort studies have proven that in anti-synthetase syndrome or inflammatory myopathy, anti-SS-A antibody-positive individuals develop more severe ILD including more extensive pulmonary fibrosis and decreased LF. Additionally, these patients are less responsive to immunosuppressive therapies (La Corte et al., 2006; Váncsa et al., 2009). Literature about the diagnostic utility of separated anti-SS-A antibodies is heterogeneous (Hervier et al., 2009; Langguth et al., 2007; Robbins et al., 2019). According to the official recommendation for IPAF by ATS/ERS, in serological domain, Ro60 and Ro52 antibodies are not separated (Fischer et al., 2015). Therefore, we analyzed mixed anti-SS-A level.

Another predictor of progression was post-exercise pulse increase at the 6MWT. The connection between heart rate and 6MWT has not been studied profoundly before in CTD-ILD and IPAF patients; however an association has been found to be a prognostic marker in IPF (Holland et al., 2013). Although, chronotropic response abnormality cannot be certainly established due to various comorbidities and medication history regarding beta blockers being inaccessible (Sanges et al., 2017). The third variable for confirmed faster progression of PF-ILD in our patients was malignancy. Malignancy as a comorbidity is a serious complication associated with ILDs, especially in those showing progression as published previously in our previous study (Barczi et al., 2020).

Defining progression is a difficult task, as for CTD patients several treatment possibilities are open for their underlying disease. According to recent studies in IPF and CTD-ILD patients, a decrease in DLCO is proposed in the definition of PF-ILD (Khanna et al., 2015; Volkmann et al., 2019; Wong et al., 2020; Cottin et al., 2018). Inclusion criteria for PF-ILD subjects in the INBUILD (Efficacy and Safety of Nintedanib in Patients With PF-ILD) trial included DLCO of at least 30% and less than 80% predicted (Brown et al., 2020; Flaherty et al., 2019). Low baseline DLCO is also a clinically meaningful risk factor for acute exacerbations (Wong et al., 2020). In our study, patients had decreased DLCO; however, we did not find any correlation between progression and DLCO change.

We provided real-world data on the treatment and functional outcome for these special patient groups. Therapy in CTD-ILD changes according to underlying disease, while no therapy guidance for IPAF is available (Sambataro et al., 2018; Gao and Moua, 2020). PF-ILD is much more of a disease phenotype than a diagnosis. Timely initiation of antifibrotic therapy slows the progression of the disease (Johannson et al., 2021). In our study, the ILD team recommended antifibrotic treatment to patients with a rapid progression and to those with IPF characteristics. More patients with IPAF and progression were offered this therapy than CTD-ILD patients showing PF-ILD phenotype, mainly due to the fact that the antifibrotic nintedanib was only approved for PF-ILD based on the data of the INBUILD trial in 2020 (Flaherty et al., 2019; Wells et al., 2020; European Medicines Agency, 2019).

Antifibrotic treatment did stabilize lung function in the majority of our patients. PF-ILD was detected in nine patients (CTD-ILD *n* = 4; IPAF *n* = 5) who did not receive antifibrotics including 44.4% with anti-SS-A positivity and 55.5% with post-exercise pulse increase, emphasizing the need for possible extension of antifibrotic treatment. Data on the effectivity of combination therapy using different immunosuppressive treatments with antifibrotics is lacking. In real life, patients under immunosuppressive or immunomodulatory therapy are not excluded from additional antifibrotic therapy. However, in the INBUILD study, restricted therapies were only applied after 6 months of deterioration (Cottin et al., 2021). Similarly, SSc-ILD treatment outcome of SENSCIS secondary analysis showed that mycophenolate mofetil and nintedanib co-treated patients did benefit the most from treatment; however, the study was not powered for combination treatment effectivity (Distler et al., 2019; Highland et al., 2021). After applying the combination of different immunosuppressive treatments with antifibrotics, two-thirds of patients experienced mild adverse events in our cohort. Safety and tolerability profile was consistent with the product label and similar to our previously published data (Barczi et al., 2019). In our patients, 67% experienced an adverse event, similar to the INBUILD trial, where diarrhea was observed in 67%, followed by nausea (29%) (Flaherty et al., 2019). The single grade 3 adverse event of liver enzyme increase needing drug discontinuation was resolved by changing to another antifibrotic agent. Acute exacerbations are serious complications of ILDs (Suzuki et al., 2020; Kolb et al., 2018). Unfortunately, our data were not available to analyze these effects on progression.

In conclusion, the majority of autoimmune-associated ILDs including CTD-ILD and IPAF might be stable or even improve due to proper combination therapy. Patients receiving antifibrotic treatment were less likely to deteriorate and fulfill criteria for PF-ILD. Progression was associated with anti-SS-A antibodies, post-exercise pulse increase at 6MWT, and concomitant malignancies—patients presenting with these parameters should be followed more closely. Antifibrotic treatment was effective in stabilizing functional decline, and the drugs confirmed a safety and tolerability profile consistent with the product label. More data is needed in a real-world setting to identify optimal combination therapies and timing for initiation of antifibrotics in CTD-ILD and IPAF patients. Stable lung function might be a result of the relatively short observation period, and more longitudinal data are awaited.

The main limitation of our study includes the retrospective single-center design and limited number of patients. Further prospective studies need to evaluate this special subgroup of ILD patients to develop guidelines for optimal treatment start and combination therapies.

On the other hand, our data are the first to represent ILD distribution of cases from an Eastern European country. Our study is based on long-term longitudinal follow-up of ILD patients with autoimmune characteristics. Disease population covered the two main rheumatology centers in the region of Central Hungary.

## Data Availability

The original contributions presented in the study are included in the article; further inquiries can be directed to the corresponding author.
